# Monitoring of deep lymphocyte phenotypes in the blood and bronchoalveolar lavage fluid of patients with severe malaria-associated acute respiratory distress syndrome

**DOI:** 10.62675/2965-2774.20250409

**Published:** 2025-07-15

**Authors:** André Miguel Carapinha Gomes, Maria Adão-Serrano, Maria Ribeiro da Cunha, João Santos Silva, Ana Espada de Sousa, João Miguel Ribeiro, Susana Mendes Fernandes

**Affiliations:** 1 Gulbenkian Institute for Molecular Medicine Lisboa Portugal Gulbenkian Institute for Molecular Medicine - Lisboa, Portugal.; 2 Hospital de Santa Maria ULS Santa Maria Lisboa Portugal Hospital de Santa Maria, ULS Santa Maria - Lisboa, Portugal.; 3 Universidade de Lisboa Faculdade de Medicina Lisboa Portugal Faculdade de Medicina, Universidade de Lisboa - Lisboa, Portugal.

**Keywords:** Malaria, Respiratory distress syndrome, Chemokine receptors, Immune phenotyping, Extracorporeal membrane oxygenation

## Abstract

Restoring immune homeostasis after an acute insult is essential for achieving a full recovery from an acute respiratory distress syndrome episode. Immune monitoring tools that are not exclusive to the blood compartment are in great demand to help guide treatment decisions. In this longitudinal study, we report a case of severe malaria-associated acute respiratory distress syndrome supported by venovenous extracorporeal membrane oxygenation. Although there was persistent lymphopenia, we observed dynamic shifts in T cells and rare innate lymphoid cell populations. The type 2 immune profile was preponderant at the acute phase, and decreased exhausted T-cell populations indicated recovery. There were significantly different blood and bronchoalveolar lavage fluid profiles. Multiple-compartment immune monitoring is possible and valuable for precise immune modulation.

## INTRODUCTION

Acute respiratory distress syndrome (ARDS) occurs due to a nonregulated immune response to several acute triggers and damages the lungs.^(1)^ The inability to rapidly restore lung homeostasis contributes to secondary infections, lung fibrosis, and high mortality.^(2)^ Immune monitoring tools, including the personalized use of corticosteroids, checkpoint inhibitors, and anti-cytokine monoclonal antibodies, are in great demand to help guide treatment decisions.^(3)^ Malaria-associated ARDS (MA-ARDS) usually occurs after parasitemia control due to the host immune response.^(4)^ Herein, we report the lymphocyte kinetic response in the peripheral blood and lung compartment in a unique case of MA-ARDS supported with venovenous extracorporeal membrane oxygenation (VV-ECMO) until complete recovery nine months later.

## CASE REPORT

**Figure 1:**
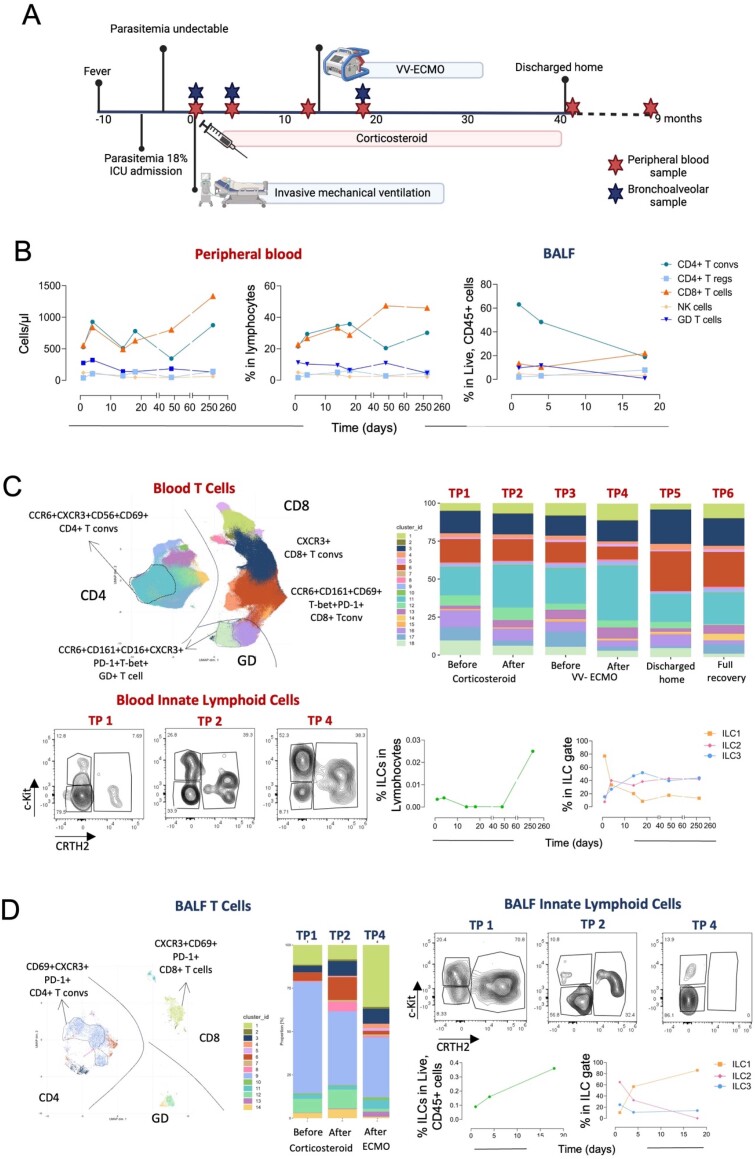
Clinical timepoints and deep immune monitoring during malaria-associated acute respiratory distress syndrome in blood and bronchoalveolar lavage fluid.

A 52-year-old man with no relevant medical history was admitted to a university tertiary hospital intensive care unit (ICU) with a five-day fever due to *Plasmodium falciparum* and presented with hyperbilirubinemia, acute kidney injury, and noncardiogenic pulmonary edema. After treatment with quinine and doxycycline for 48 hours, parasitemia resolved (Figure 1A). Nevertheless, gas exchange deterioration led to noninvasive ventilation on day 6 and invasive mechanical ventilation on day 10 of symptoms (Timepoint [TP] 1). The patient then developed severe MA-ARDS, which was treated with dexamethasone and later with methylprednisolone due to concomitant organizing pneumonia. His clinical course was complicated by ventilator-associated pneumonia with secondary bacteremia due to *Klebsiella pneumoniae* and refractory hypoxemia with acidemia requiring VV-ECMO rescue (Avalon Elite® Bi-Caval Dual Lumen Catheter) for 18 days on day 21 after malaria diagnosis. He was discharged home on oxygen therapy and corticosteroids after a hospital stay of 42 days. After nine months, the patient fully recovered, and the steroid treatment was weaned entirely (TP6).

(A) Clinically relevant events after malaria diagnosis. Lengths of invasive mechanical ventilation, venovenous extracorporeal membrane oxygenation support, and corticosteroid treatment are indicated by the corresponding bars; (B) Graphs showing the frequency and number of principal cell types in peripheral blood (left) and in bronchoalveolar lavage fluid (right) at each timepoint; (C) Blood T and innate lymphoid cells. In the upper left part, uniform manifold approximation and projection with overlaying FlowSOM clusters of blood T cells are shown. On the right, the proportion of each cluster in the various timepoints is shown. In the lower left part, contour flow cytometry plots of innate lymphoid cells at timepoints 1, 2 and 4; on the right, the graphs show the frequencies of innate lymphoid cells in total lymphocytes and of each subset in an innate lymphoid cell gate at different timepoints; (D) Bronchoalveolar T and innate lymphoid cells. Uniform manifold approximation and projection with overlaying FlowSOM clusters of bronchoalveolar lavage fluid T cells and the proportion of each cluster at the three timepoints (left); (right, upper) contour flow cytometry plots of bronchoalveolar lavage fluid innate lymphoid cells at timepoints 1, 2 and 4 and (right, lower) frequency of the principal subsets at the three timepoints.

ICU - intensive care unit; VV-ECMO - venovenous extracorporeal membrane oxygenation; BALF - bronchoalveolar lavage fluid; GD - gamma delta; TP - timepoint.

Peripheral blood and bronchoalveolar lavage fluid (BALF) samples were longitudinally obtained (Figure 1A) upon clinical request, and high-dimensional spectral flow cytometry tubes with 21 colors were used to subset both T cells and innate lymphoid cells (ILCs).

Lymphopenia was a hallmark until complete recovery at TP6 (Figure 1B). Compared with TP6, timepoint 1 presented a lower number of CD4 (468 *versus* 1014 cells/µL) and CD8 (467 *versus* 1332 cells/µL) T cells but an absolute increase in gamma-delta+ (GD) T cells (237 *versus* 121 cells/µL) and NK cells (107 *versus* 61 cells/µL). Blood expansion of effector GD+ T-cell populations, particularly clusters 18 and 16, was observed (Figure 1C). Throughout the more severe phase, until TP4, there was a sustained increase in the proportion of a specific population of CD4+ T cells (CCR6+CXCR3+CD69+ cells) in the blood. Interestingly, in the BALF, parallel accumulation of CD4+ T cells expressing high levels of CD69, PD-1 and CXCR3 was observed, suggesting activated resident Th1 cells.

There were significant shifts in the T-cell composition of the BALF between TP1 and TP4 (CD4+ conventional: 63.1 *versus* 18.9%; GD: 9.6 *versus* 0.9%; CD8: 13.2 *versus* 21%; CD4+ regulatory: 2.0 *versus* 7.8%; Figure 1D). We documented an influx of ILC2s into the lung in the early phase, eventually reflecting the malaria response, followed by an increase in the number of ILC1s.

## DISCUSSION

This was the first study to report the detailed immune lymphocyte profile of a patient with MA-ARDS. Our longitudinal findings included data collected between the initiation of mechanical ventilation and complete recovery nine months later. The results provide support for the hypothesis that severe disease is characterized by ILC2 and GD T-cell pulmonary infiltration, and recovery is characterized by decreased exhausted/activated CD4+ and CD8+ T-cells.

In the early phase, gamma delta expansion might contribute to initial lung lesions, which decline early after corticosteroid treatment (from TP2 to TP4). Additionally, the hyperacute phase was characterized by an expansion of CXCR3+ T-cells. Notably, blocking CXCR3 was found associated with decreased organ lesions and mortality in a sepsis model.^(5)^ Later in the disease process, the increase in regulatory T-cells (Tregs) in parallel with a change in the ILC profile might be relevant for lung recovery.^(6)^ Although they represent rare populations, ILCs are fundamental to lung repair, as we have previously documented in patients with severe COVID-19, where recovery was linked with an increase in ILC1s.^(7)^

## CONCLUSION

Complex immune monitoring is fundamental for better understanding acute disease phenotypes and trajectories, as well as the response/impact of immune modulator drugs (e.g., corticosteroids or targeted therapies).

## References

[1] Huang Q, Le Y, Li S, Bian Y (2024). Signaling pathways and potential therapeutic targets in acute respiratory distress syndrome (ARDS). Respir Res.

[2] Grasselli G, Calfee CS, Camporota L, Poole D, Amato MB, Antonelli M (2023). European Society of Intensive Care Medicine Taskforce on ARDS. ESICM guidelines on acute respiratory distress syndrome: definition, phenotyping and respiratory support strategies. Intensive Care Med.

[3] Serrano MA, Gomes AM, Fernandes SM (2022). Monitoring of the forgotten immune system during critical illness-a narrative review. Medicina (Kaunas).

[4] Mukherjee D, Chora ÂF, Lone JC, Ramiro RS, Blankenhaus B, Serre K (2022). Host lung microbiota promotes malaria-associated acute respiratory distress syndrome. Nat Commun.

[5] Herzig DS, Guo Y, Fang G, Toliver-Kinsky TE, Sherwood ER (2012). Therapeutic efficacy of CXCR3 blockade in an experimental model of severe sepsis. Crit Care.

[6] D’Souza SS, Shen X, Fung IT, Ye L, Kuentzel M, Chittur SV (2019). Compartmentalized effects of aging on group 2 innate lymphoid cell development and function. Aging Cell.

[7] Gomes AM, Farias GB, Dias-Silva M, Laia J, Trombetta AC, Godinho-Santos A (2021). SARS-CoV2 pneumonia recovery is linked to expansion of innate lymphoid cells type 2 expressing CCR10. Eur J Immunol.

